# Drug Use, Family Support, and Depressive Symptoms Among Latinx Sexual Minority Men: A Longitudinal Analysis

**DOI:** 10.1007/s10461-023-04098-w

**Published:** 2023-06-12

**Authors:** Homero E. del Pino, Nina T. Harawa, Steven J. Shoptaw, Katrina Schrode, Arun Karlamangla

**Affiliations:** 1 Charles R. Drew University of Medicine and Science, 1731 E. 120th St, Los Angeles, CA 90059, USA; 2 David Geffen School of Medicine at UCLA, Los Angeles, CA 90095, USA; 3 VA Greater Los Angeles Healthcare System (Geriatric Research, Education, and Clinical Center), Los Angeles, CA 90073, USA

**Keywords:** Latinx/Latino, Sexual Minority Men, Family Support, Drug Use, Mental Health

## Abstract

Family rejection has negative health consequences for Latinx sexual minority men (LSMM). However, LSMM often reconcile with their families, a phenomenon cross-sectional studies miss. We analyzed longitudinal data from the Healthy Young Men’s Study in Los Angeles. We used individual fixed-effects Poisson regression to estimate associations among within-individual changes over time in family support, drug use, and depressive symptoms. We found that (1) initiation of drug use was associated with 7.2% increase (Ratio= 1.072, 95% CI 1.006 – 1.142, p= 0.03) in family support among LSMM who reported high depressive symptoms (depression subscale T-score ≥ 63) in at least one data wave; (2) 1-unit increase in family support was associated with 4.7% decrease (RR = 0.953, 95% CI 0.931 – 0.976, p< 0.001) in the probability of high depressive symptoms; and (3) no significant association between change in drug use and change in high depressive symptoms. Over time, LSMM appear to benefit from the health effects of family support associated with Latinx family structures.

## Introduction

Latinx familism is associated with positive physical and mental health outcomes [1–7]. Latinx family life is qualitatively distinct from that of non-Hispanic Whites [8]. It differs in at least three dimensions: structural (presence of nuclear and extended kin), behavioral (provision of social support), and attitudinal (prioritizing family loyalty and solidarity among members) [9, 10]. Another feature of Latinx familism is that members often choose healthy behaviors to “do right” by their family [11]. Hence, familial cultural values have been leveraged to reduce drug and alcohol use among Latinx youth [12–14]. Among adolescents, familism is associated with fewer depressive symptoms [15, 16], while family conflict is associated with higher levels of depressive symptoms [17].

However, Latinx sexual minority men (LSMM) do not seem to reap all the health benefits of familism. Compared to the parents of White SMM, parents of LSMM tend to have more traditional gender role beliefs that are associated with higher levels of rejection of their son’s sexual orientation [18]. Rejection has negative health consequences: LSMM who reported higher levels of family rejection during adolescence were 5.9 times as likely to report high levels of depressive symptoms and 3.4 times as likely to use psycho-active drugs compared with those who reported no or low levels of family rejection [19]. Some LSMM report using drugs to cope with family rejection and stigma [20, 21]. A study of over 900 LSMM across three cities in the United States found that 80% of them reported having depressed mood [20]. Mental health challenges among Latinx adolescents and adults are associated with drug use [22, 23].

Family rejection is not the whole story for LSMM. After coming out, many LSMM reconcile with their families over time, a phenomenon that cross-sectional studies miss [24, 25]. We know that family dynamics and behaviors also change over time, so we were interested in the temporal trends in the associations between family support and both drug use and depressive symptoms. We focus on drug use and depressive symptoms because people who use drugs and report symptoms of depression are more likely to exhibit social dysfunction and social impairment than people who use drugs but report no symptoms of depression [26, 27]. Hence, LSMM who report both drug use and depressive symptoms may exhibit more social dysfunction, signaling a need for greater familial support than those who use drugs but do not also have a mental health disorder.

Most research has focused on cross-sectional associations between family support or rejection and drug use and mental health. In contrast, we analyzed longitudinal data of young LSMM in Los Angeles to examine changes *over time* in the associations among family support, drug use, and mental health. For drug use, we focused on stimulants and opioids because these are two classes of drugs that are common to young adults who hold sexual minority identities in Los Angeles and are linked to health threats, including depressive symptoms [28, 29]. We controlled for living situation because living situations structure social relationships, the process of social support, and the self-management behaviors of individuals [30, 31]. We tested three hypotheses:

### H1: Over time, increases in drug use lead to an increase in the level of family support.

We tested whether an increase in reported drug use over time led to a concurrent increase in family support among people who reported high depressive symptoms in at least one wave of the study. We restricted the analysis to this group because, in those not prone to depressive symptoms, families may not become aware of their drug use because their use has likely not impacted their ability to function [26, 27]. To confirm this potential difference between the groups, we also separately tested for a relationship between increase in drug use and increase in family support among the group of participants who never reported depressive symptoms in the study, although we did not expect to see a significant association.

### H2: Increases in the level of family support over time lead to concurrent decreases in the probability of high depressive symptoms (defined as depression subscale T-score ≥ 63).

We examined whether increases in family support over time in those who reported depressive symptoms in at least one wave led to a concurrent decrease in depressive symptoms.

### H3: Over time, increases in reported drug use lead to a decrease in the probability of high depressive symptoms (defined as depression subscale T-score ≥ 63).

If an increase in drug use over time leads to an increase in family support, which in turn leads to a decrease in depressive symptoms, we should also see a relationship between an increase in drug use and decrease in depressive symptoms. Because this could play out over a longer period than 6 months (the time between successive study visits), we tested for both a concurrent relationship between increase in drug use and decrease in depressive symptoms and a lagged relationship (i.e., decrease in depressive symptoms one study visit after the increase in drug use) in separate models.

## Methods

### Overview

This study analyzed data from the Healthy Young Men’s Study (HYM), a longitudinal research project focused on preventing HIV and improving the health and wellness of Black, Latinx, and multiracial young men who have sex with men in Los Angeles. Data are collected in the following domains: insurance status and access to care; drug use including alcohol, marijuana, and other illicit drugs; use and access to HIV/STI testing and treatment services; for young men with HIV, their retention in HIV/AIDS care and adherence to anti-retroviral treatment; and use of biomedical prevention interventions, such as pre-exposure prophylaxis (PrEP) and post-exposure prophylaxis (PEP). (For a full description see Kipke et al. [32].)

### Description of Data

#### Original Data Source

A total of 448 subjects were recruited into the HYM study. Young men were eligible to participate if they were assigned male sex at birth; 16 to 24 years old; self-identified as gay, bisexual, or uncertain about their sexual orientation; reported having had sex with another man within the last 12 months; a resident of Los Angeles or a surrounding county and did not anticipate moving for at least six months; and self-identified as African American/Black or Hispanic/Latino or multi-racial/ethnic. The young men were recruited using both venue-based and social media recruitment strategies [33].

#### Analytic Sample

We restricted our sample to the 291 participants who self-identified as male and Hispanic/Latino, regardless of race. We analyzed six waves of data collected between 2016 and 2020.

## Measures

### Outcomes

#### Perceived Social Support from Family (α = 0.90) [34].

We used four items from this study instrument (e.g., I can talk about my problems with my family). Responses were given on a 7-point Likert scale ranging from 1-”Very Strongly Disagree” to 7-”Very Strongly Agree.” We calculated the total family support score as the sum of the 4 items. Higher scores reflect greater levels of perceived social support from family members.

#### Depressive Symptoms [35].

We used the Brief Symptom Inventory 18 to measure psychological distress over the past seven days. It consists of 18 items on a 5-point (0–4) Likert scale and includes subscales for somatization, depression, and anxiety. We focused on the depression subscale (α = 0.84), which consists of 6 items. A high score indicates greater distress. The maximum raw score for this subscale is 24; scores were converted to a T-score based on community norms. We dichotomized the variable, < 63 or ≥ 63; a T-score of 63 indicates a higher level of depressive symptoms.

### Predictors

#### Drug Use.

We focused on stimulants and opioid use. We defined this variable as self-reported use of stimulants (cocaine, crack, methamphetamine) or opioids (heroin, fentanyl) in the last 6 months. We created a dichotomous variable (yes/no) for reported use of any of these drugs due to the heterogeneity of drugs reported and the small numbers in cells for specific drugs used.

#### Living situation.

This was a categorical variable to indicate where participants lived during each wave of the study: with family, with friends/partner, own place, or no regular place.

## Analysis Plan

We used individual fixed-effects (IFE) Poisson regression to estimate associations among within-individual changes over time in the outcomes as a function of changes over time in the predictors listed above. We chose IFE regression to maximize our ability to infer causality because IFE regression eliminates confounding by *all* time-invariant characteristics of the participant, regardless of whether they are measured (such as ethnic origin) or not measurable (e.g., inherited tendencies). IFE models can be thought of as ordinary linear regression with separate flags for each individual participant included as covariates. To control for time-varying characteristics, we included living situation in a given wave (with family, own place, with friends/partner, no regular place) as a covariate in the IFE models.

We used the *fixest* package in R to conduct the analyses; we used the standard 5% threshold for Type I error (alpha) to determine statistical significance.

## Results

### Participant Characteristics

The mean age of participants at baseline was 22.1 (SD = 2.0) and across waves was 23.3 (SD = 2.0). Seventy-four (25%) reported living with their families across all waves; 141 (49%) were employed across all waves; and 58 (20%) were in school across all waves. Only 13 (5%) participants reported depressive symptoms across all waves (depression subscale T-score ≥ 63). Nearly half the cohort, 140 (48%), reported high depressive symptoms at least once, while 151 (52%) participants did not report any depressive symptoms at any wave (Table I). The mean level of family support at baseline was 15.4 (SD = 5.7) on a 4–28 point scale.

We also found a small difference in the pattern of drug use between those who never reported high depressive symptoms in any wave and those who reported high depressive symptoms in at least one wave. This finding held both at baseline and across waves (Table II).

### Multivariable, Individual Fixed Effects Regression Analysis

#### H1: Over time, increases in drug use lead to an increase in the level of family support.

Initiation of drug use was associated with 7.2% increase (Ratio= 1.072, 95% CI 1.006 – 1.142, p= 0.03) in family support among LSMM who reported high depressive symptoms (depression subscale T-score ≥ 63) in at least one data wave; no association (Ratio = 1.014, 95% CI 0.964–1.067, p = 0.57) was found among those who never reported high depressive symptoms; and living situation had no association with family support in either group of participants (Table III).

#### H2: Increases in the level of family support over time lead to concurrent decreases in the probability of high depressive symptoms.

A 1-unit increase in family support was associated with 4.7% decrease (RR = 0.953, 95% CI 0.931 – 0.976, p< 0.001) in the probability of high depressive symptoms (depression subscale T-score ≥ 63) (Table IV).

#### H3: Over time, increases in reported drug use lead to a decrease in the probability of high depressive symptoms (defined as depression subscale T-score ≥ 63).

Contrary to our hypothesis that a within-person change in drug use was predictive of a within-person change in the probability of high depressive symptoms occurring either concurrently or delayed, we did not find any significant association between change in drug use and change in high depressive symptoms (Table V).

## Discussion

Family support can play a positive role in the health of LSMM. In Hypothesis 1 we tested whether uptake of drug use over time led to an increase in the level of family support. We found that over time, initiating drug use is associated with a 7.2% (Ratio = 1.072, 95% CI 1.006–1.142, p = 0.03) increase in the participants’ perceived level of family support only for those who reported high depressive symptoms in at least one wave. This finding suggests that families play an important role in addressing drug use among LSMM and aligns with studies that show that familial cultural values are important to address drug use [12–14]. Conversely, stopping drug use is associated with a decrease in perceived family support. This may be because participants do not require a greater level of support once they stop their drug use. Because family support increased only among participants who reported high depressive symptoms in at least one wave, we infer that families may need more education to identify problematic drug use in the absence of depressive symptoms to be able to support these individuals as well.

In Hypothesis 2 we tested whether, over time, increases in the level of family support lead to a decrease in the probability of high depressive symptoms (defined as depression subscale T-score ≥ 63). Findings support this hypothesis, with increases in family support related to a 4.7% (RR = 0.953, 95% CI 0.931–0.976, p < 0.001) decrease in the probability of high depressive symptoms among participants who reported such symptoms in at least one wave. This is consistent with studies showing that familism is associated with decreased depressive symptoms [15, 16]. For LSMM facing mental health challenges, family support makes a difference. Juxtaposing this finding with studies demonstrating the negative links between family rejection and mental health outcomes [17, 19, 20] challenges us to develop nuanced understandings of how family ties affect the mental health of LSMM beyond initial experiences of rejection of their sexual orientation.

Because reported drug use initiation was associated with an increase in the level of family support, and an increase in the level of family support was associated with a decrease in the probability of high depressive symptoms, we tested Hypothesis 3, that over time, increases in reported drug use lead to a decrease in the probability of high depressive symptoms (defined as depression subscale T-score ≥ 63). We found no associations. Although families are protective against drug use and depressive symptoms, their support does not guarantee that their members will not develop a substance use disorder or a mental health disorder. The long-term effect of drug use on family support and mental health among LSMM requires further study.

The majority of participants (74.6%) did not live with their families of origin at all waves. Yet there was no statistically significant difference between living at home or having other living arrangements in terms of family support and depressive symptoms outcomes. Further research is needed to determine whether the living situation of LSMM moderates the association between levels of family support and drug use initiation and the association between family support and high depressive symptoms. Exploring living situation as a moderator of family support could complement studies showing that Latinx people report greater familial support than other racial/ethnic groups and are more likely to live with extended family [6, 36].

This study points to potential future studies on LSMM, drug use, and family support. Our results imply that we need longitudinal research on how the family members of LSMM can be engaged in drug use interventions. First, some LSMM with a history of drug use have reported that strained family ties were reestablished over time [24, 25]. Second, some LSMM with a history of drug use have reported that they prioritize family support for behavior change despite experiences of family rejection [19, 20, 24, 37]. Hence, a nuanced view of family support over time may help us overcome the dearth of family-based drug use interventions specifically for LSMM. To our knowledge, most Latinx family-based interventions for drug use have overlooked SMM, even though connectedness with family protects against drug use in this and other populations of young Latinx people [38–41].

We speculate that more interventions to promote acceptance of sexual orientation among family members are needed. Some LSMM may never experience acceptance from their families or it may take decades [42], while others do experience varying levels of acceptance [24]. We need to understand the processes by which some Latinx families come to accept LSMM’s sexual orientation. This may be accomplished by interviewing LSMM about their familial interactions after their families learned of their sexual orientation. At the same time, we need to ensure we develop a culturally and generationally appropriate understanding of what acceptance of sexual orientation looks like within Latinx families [24]. This may be done by interviewing family members about their own journey of acceptance of their LSMM relative.

## Limitations

We do not know whether the participants were out to their families before or during the study, or not at all. This could affect family support. We also do not know whether and to what extent LSMM in our sample experienced family rejection and resolution related to their sexual orientation. However, unlike cross-sectional studies, our study found that over time, increases in drug use are associated with increases in family support, which in turn are associated with a decrease in depressive symptoms at potentially critical points in the lives of young LSMM. The association between increases in drug use and increases in family support suggests that the effect of families on LSMM’s mental health and their relevance for addressing drug use is dynamic and important [24].

## Conclusion

Despite potential experiences of family rejection, LSMM over time appear to benefit from the health effects of family support associated with Latinx family structures. Our findings suggest that families can play a significant role in helping to bring about positive health outcomes for LSMM. Conducting longitudinal analyses of family support of LSMM demonstrates that family rejection does not represent the full experience of LSMM family life. This is missing in our conversations about LSMM. Ultimately, gaining a deeper and more subtle understanding of the role of family support over time in the lives of LSMM may help us address their drug use and mental health challenges.

## Figures and Tables

**Table I:** Characteristics of participants across all waves (*n* = 291)^a^

	Mean	SD
**Age**	23.3	2.0
	*n*	%
**Ethnic origin**		
Mexican	120	41.2
Central American	30	10.3
South American	5	1.7
Caribbean	11	3.8
Multiple origins	125	42.9
**HIV status**		
Negative	245	84.2
Positive	31	10.7
Sero-converted	15	5.2
**Living with family at every wave**		
No	217	74.6
Yes	74	25.4
**In school at every wave**		
No	233	80.1
Yes	58	19.9
**Employed at every wave**		
No	150	51.6
Yes	141	48.5
**Ran out of money for basic needs at any wave**		
No	118	40.6
Yes	173	59.5
**High level of depressive symptoms at every wave**		
No	278	95.5
Yes	13	4.5
**High level of depressive symptoms at any wave**		
No	151	51.9
Yes	140	48.1
**Reported use of stimulants or opioids at any wave**		
No	166	57.0
Yes	125	43.0
**Changes in stimulant or opioid use at any wave**		
Initiated at any wave	55	19.0
Stopped at any wave	47	16.3

a Percentage totals may not equal 100% for some variables due to rounding.

**Table II:** Changes over time in drug use in those who never reported high levels of depressive symptoms and in those who reported high levels of depressive symptoms in at least one wave

	Drug use at Baseline		New use at a follow-up		No change in any follow-up		Quit using at a follow-up	
	*n*	%	*n*	%	*n*	%	*n*	%
**Never reported high levels of depressive symptoms (N = 151)**	32	21.2	60	9.9	65	10.7	57	9.4
**Reported high levels of depressive symptoms in at least 1 wave (N = 140)**	38	27.1	62	10.4	101	17.0	60	10.1

Baseline: *X*^*2*^(1) = 1.41; p = 0.23

**Table III:** Change in drug use as a predictor of change in level of family support^a^

	Reported high depressive symptoms ≥ 1 wave			Never reported high depressive symptoms		
	Ratio	95% CI	p-value	Ratio	95% CI	p-value
**Drug use**	1.072	1.006–1.142	**0.03**	1.014	0.964–1.067	0.57
**Living situation**						
with family	**Referent**			**Referent**		
own place	1.014	0.942–1.091	0.71	0.988	0.936–1.043	0.66
with friend/partner	0.996	0.880–1.127	0.95	0.912	0.814–1.021	0.11
no regular place	1.015	0.912–1.129	0.78	1.001	0.912–1.098	0.98

a Results of individual fixed effects regression to test Hypothesis 1.

**Table IV:** Family support as a predictor of high depressive symptoms in participants who report high depressive symptoms in at least one wave^a^

	RR	95% CI	p-value
**Family support**	0.953	0.931 – 0.976	**< 0.001**
**Living situation**			
living with family	**Referent**		
own place	0.892	0.623 – 1.278	0.53
with friends/partner	0.963	0.658 – 1.410	0.84
no regular place	0.859	0.533 – 1.383	0.53

a Results of individual fixed effects regression to test Hypothesis 2.

**Table V:** Concurrent and lagged drug use and high depressive symptoms among participants who reported high depressive symptoms in at least one wave^a^

	Concurrent			Lagged		
	RR	95% CI	p-value	RR	95% CI	p-value
**Drug use**	1.033	0.771–1.383	0.83	0.864	0.601–1.241	0.42
**Living situation**						
with family	**Referent**			**Referent**		
own place	0.889	0.623–1.268	0.51	0.776	0.487–1.236	0.28
with friends/partner	0.896	0.618–1.301	0.56	0.693	0.419–1.149	0.15
no regular place	0.822	0.511–1.324	0.42	0.853	0.474–1.534	0.59

a Results of individual fixed effects regression to test Hypothesis 3.

## Data Availability

The data analyzed during the current study are available from Michele Kipke, PhD, principal investigator of the Healthy Young Men Study, on reasonable request.
